# Periprosthetic fractures around the knee—the best way of treatment

**DOI:** 10.1007/s12570-012-0130-x

**Published:** 2012-08-21

**Authors:** Steffen Ruchholtz, Jordi Tomás, Florian Gebhard, Morten Schultz Larsen

**Affiliations:** 1 Department of Trauma, Hand and Reconstructive Surgery, University Hospital Marburg, Baldingerstrasse, 35043 Marburg, Germany; 2 Trauma Unit CSU Vall d´Hebron Barcelona, Barcelona, Spain; 3 Department of Trauma, Hand and Reconstructive Surgery, University Hospital Ulm, Ulm, Germany; 4 Department of Orthopaedics, Trauma section, Odense University Hospital, Odense, Denmark

**Keywords:** Locking plate, Polyaxial locking plate, Periprosthetic knee fracture, Minimally invasive surgery, Mini open surgery, Revision arthroplasty

## Abstract

**Background:**

A variety of methods has been described to stabilise periprosthetic fractures around total knee arthroplasty (TKA). Our report offers a review of the actual strategies in the reduction and fixation of these fractures. Surgical treatment should be based on the following four steps:

1. Diagnostics: By taking the patients' history together with an X-ray of the knee and femur, the fracture is analysed. It is crucial to define whether any losening of the prosthesis had occurred. In selected cases, CT-scan may add important information on the stability of the implant.
2. Classification and planning: For most fractures around the distal femur, the Rorabeck classification is used while fractures around the proximal tibia are best classified according to the Felix classification. Additionally the Orthopaedic Trauma Association (OTA) may be helpful in the planning process for reduction and fixation.
3. Surgigal technique: In fractures around a stable implant (Rorabeck type I and II; Felix type A and C), it is favourable to use plates and retrograde nails (in Rorabeck I or II with an open box of a TKA). For reduction, three methods are available: (a) the open technique (with direct or indirect reduction); (b) the mini open technique (direct reduction of the fracture by cerclage or lag screw and percutaneous plate fixation in OTA type 32 or 33-A1) and (c) the minimally invasive technique (indirect reduction and percutaneous fixation in all other OTA types). Fractures with a loose prosthesis (Rorabeck III and Felix B) are best stabilised by hinged revision arthroplasty.
4. Rehabilitation: It is of great importance for the aged patient to be mobilised out of bed early. In most of the cases, partial weight bearing has to be performed by the aid of frames during the first 6 weeks after surgery. In a well-fixed revision prosthesis with a cemented stem, early full weight bearing might be allowed.

**Conclusion:**

Standardised less invasive procedures to treat periprosthetic fractures present a valuable alternative to open techniques. The main advantages are lower rates of oft tissue complications and implant failures following less invasive techniques of long plate application. Polyaxial locking systems allow for stable plate fixation around intramedullary implants.

## Introduction

In ageing societies, the demand for joint arthroplasty repairing osteoarthritis in the proximal and distal femur continually increases. In Germany, a total of about 450,000 primary hip and knee arthroplasties due to arthrosis are performed every year [1, 2]. At the same time, there is a growing incidence of revision surgery after joint replacement. About 10,000 revisions of knee arthroplasties are performed, with an annual increase of about 10 % in Germany caused by the growing number of patients with long standing implants [1]. Periprosthetic fractures (PPF) after knee arthroplasty occur in up to 2.5 % mounting up to 38 % after revision surgery [3–7].

The localization of PPF less frequently concerns the tibial component than the distal femur amounting up to 4 % of the cases. Felix showed in his work in 1997 that 19 % of these periprosthetic (PP) tibial fractures are brought forth intraoperatively during implantation of the prosthesis [8].

Important complication rates of up to 41 % and revision rates of 29 % of cases are recently reported for surgical treatment of periprosthetic fractures [9–14].

Various reasons may eventually lead to postoperative problems:

1. Bone quality is poor due to pre-existing osteoporosis
2. Stable fixation is difficult to achieve in areas with an intramedullary implant
3. Fracture healing is significantly delayed in aged patients
4. Prosthesis loosening may facilitate the resulting fracture

Typical complications after internal fixation of periprosthetic fractures are the following:

1. Loss of fixation
2. Non-union and implant failure
3. Infection
4. Malrotation and malunion
5. Loosening of the prosthesis

Within this paper, we would like to present and discuss the modern strategies in classification, planning and treatment of periprosthetic fractures around knee arthroplasty.

## Femur

To date, a variety of strategies for periprosthetic fracture fixation is described in the literature. The results of conventional non locking implants have generally been poor, with complication rates up to 53 % [15]. Therefore, locking plates have become more commonly used in complicated osteoporotic fractures. Although fixation is tighter, the complication rates of this technique, nevertheless, remain high with up to 29 % failures [9–14]. The development of polyaxial screws and anatomic (periprosthetic) plates might be an advantage in improving fixation of PPF fixation [16–18].

In order to preserve the blood supply of the bone and to diminish local soft tissue complications, minimally invasive strategies are recommended by some authors even for periprosthetic fractures [11, 13, 14]. Other authors still recommend the open approach [9, 10, 12, 16, 19]. Although the revision rates in the papers describing minimally invasive techniques (0–12.5 %) seem to be lower than in studies on open approaches (7–29 % revisions), direct comparison is not possible, because fracture types, treatment strategies and implants are different.

Nevertheless, MIS strategies in PPF fixation remain challenging with respect to reduction and fixation techniques. Within this publication we will give additional tips on the less-invasive techniques in the fixation of PPF.

### Diagnostics

Standard anteroposterior and lateral views are the basis of fracture analysis and classification. Before classifying the fracture, the stability of the prosthesis should be assessed with respect to the patients’ history before the trauma (a history of pain around the prosthesis may indicate previous loosening) and the situation evaluated on conventional X-ray. Typical signs of loosening like displacement of the shield from the distal femur or separation of cement may be detected. In ambiguous cases, a CT-scan may help find signs of loosening, particularly around the femoral component (Figs. 14 and 15).

It is not always possible to establish a definitive diagnosis of a loose implant since, particularly, in a fracture situation, the loosening may be merely partial. Therefore, the surgeon should be prepared to eventually change to a revision prosthesis when an osteosynthesis was initially planned.

Sometimes, septic loosening may precede the PPF. Therefore, a microscopic (microbiological) analysis of the intraarticular fluid for white blood cells and bacteria is recommended in patients with clinical signs of infection.

### Classification

Periprosthetic fractures in the distal femur generally are classified by the Rorabeck classification [20]. The Rorabeck classification discerns loose and unloose implants according to the level of displacement. Although other classifications considering the quality of the bone stock [21] or the underlying implant (component with or without stem) have been published, their superiority still has to be proven.

The main advantage of the Rorabeck classification is its simplicity, but it is not always the best treatment guide, because other factors like the quality of the bone stock (e.g. osteoporosis), the underlying implant and the fracture type have to be considered when planning fracture fixation or replacement. In Figs. 7 and 10, it is shown how fractures that are classified as Rorabeck type II can differ with respect to the type of prosthesis and type of fracture.

In order to plan the surgical approach, the Orthopaedic Trauma Association (OTA) classification of femur fractures (no. 32 and 33) may additionally be applied (see ‘Surgical techniques’).

#### Rorabeck I

Undisplaced fractures (less than 5 mm of displacement and less than 5° of angulation) around a stable fixed prosthesis in the distal femur are classified Rorabeck I.

Historically, periprosthetic Rorabeck I fractures have been treated conservatively. Some authors have reported good results in terms of fracture consolidation following conservative methods, but, in the majority of cases, their results don’t report any data regarding knee function or systemic patient complications after the conservative treatment [22, 23].

In the 1970s to 1980s, the results of surgical management of these fractures with invasive techniques and conventional systems for osteosynthesis were, in many cases, inferior to conservative treatment. Therefore, conservative management was generally recommended for this type of fractures.

To date, both the improvement of implants (retrograde locked nails and locked plates) and the development of less invasive surgical techniques have substantially reduced surgical complication rates [24].

**Fig. 1:**
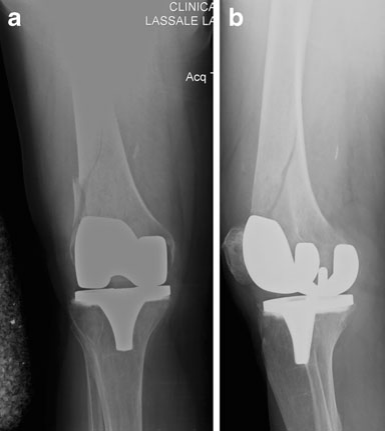
Anteroposterior (**a**) and lateral (**b**) view of a Rorabeck type 1 and OTA 33-A1 fracture around a resurfacing TKA; by Jordi Thomas

**Fig. 2:**
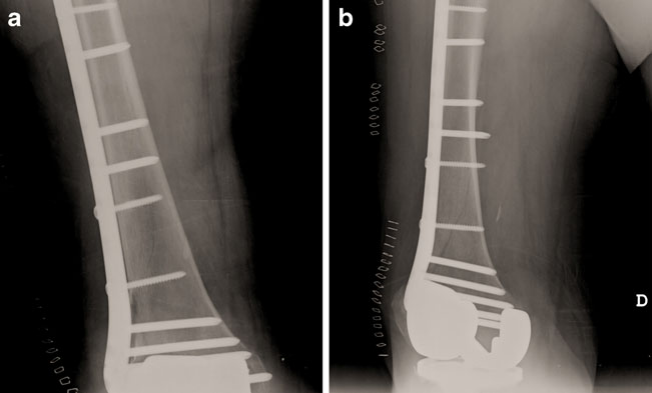
Postoperative anteroposterior (**a**) and lateral (**b**) view after mini open fixation with lag screws (through the plate) and a monoaxial locking plate (LISS Synthes®)

Because of the high risk of secondary displacement and other complications, along with the need for early mobilisation, nonoperative treatment of periprosthetic fractures of the femur may not be favourable in most patients [15] (see Figs. 1 and 2).

#### Rorabeck II

In this category, all fractures with a stable prosthesis but dislocated fragments are summarised. The treatment of choice is an open or closed reduction and internal fixation by plate or nail. Nevertheless, a revision prosthesis (see Rorabeck III) can be a choice in selected cases of very low fractures and/or severe osteoporosis.

#### Rorabeck III

Fractures around a loose prosthesis with an undisplaced or displaced fracture situation are assigned to this category. Rorabeck III fractures require a prosthetic replacement with stable fixation of the stem in the central part of the femur.

### Implants

Among a variety of possible implants, nails or plates are mostly used for the fixation of fractures with a stable prosthesis (Rorabeck I and II). In Rorabeck III fractures, the exchange to a prosthesis with a longer stem, providing proximal diaphyseal fixation, is the treatment of choice.

#### Nails

For intramedullary fixation of periprosthetic distal femur fractures, retrograde nails are used. Antegrade nails are not recommended, because distal fixation of these nails is not reliable.

**Fig. 3:**
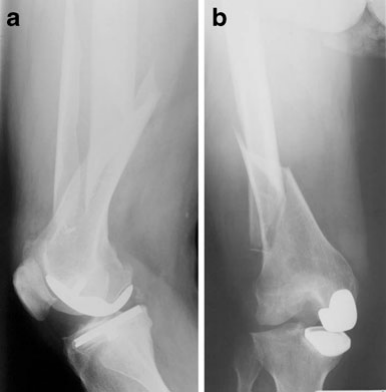
Anteroposterior (**a**) and lateral (**b**) view of a Rorabeck type 2 and OTA 32-B2 fracture around a monolateral TKA in a 77-year-old female patient; by Florian Gebhard

**Fig. 4:**
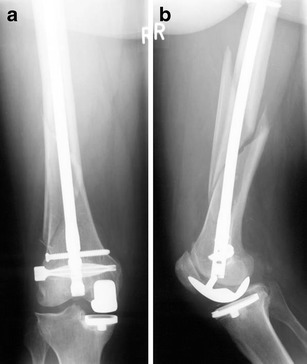
Posoperative anteroposterior (**a**) and lateral (**b**) views after minimally invasive stabilisation with an intramedullary nail (DFN with twisted plate; Synthes®). The lateral view shows a mild retroversion of the distal fragment

Before planning a retrograde nail osteosynthesis, it has to be proven that the distal entry point between the condyles of a resurfacing total knee arthroplasty (TKA) is ‘open’ (Figs. 3 and 4). In prosthesis with a box (e.g. posteriorly stabilised) or with a stem, a plate should be preferred. According to a recent analysis of the literature, there seems to be no difference in clinical outcome using a nail or a plate [15].

### Surgical technique

For surgery, the patient is placed in the supine position on a radiolucent fracture table. It has to be considered that the knee should be flexed to 90° to allow the nail to pass behind the femoral shield. Reduction and fixation is performed after a mini open or minimally invasive reduction (see: ‘Plates—Surgical technique’). For retrograde nailing, the following tips could be respected:

1. To allow optimal proximal fixation, the nail has to be long enough to pass the isthmus of the femoral intramedullary canal.
2. Distal fixation of the nail is preferably done by locked bolts or a locked twisted plate (e.g. distal femur nail; Synthes®; Figs. 3 and 4).
3. Proximal locking should be performed after distal fixation of the nail. Only thereafter the jig can be removed and the knee extended. Before proximal locking, the optimal rotation of the femur has to be evaluated.

#### Plates

Plates may be applied in nearly all PPF situations. Because of the concomitant osteopenia or osteoporosis, locking plates should be applied. The authors suggest that at least four locked screws (eight cortices) should be set in both the diaphyseal and the metaphyseal area of the femur. If this cannot be achieved with all four screws because of an intramedullary prosthetic stem or box, additional techniques (like cerclage or a locking attachment plate; Synthes®) are needed.

##### Monoaxial locking plates

**Fig. 5:**
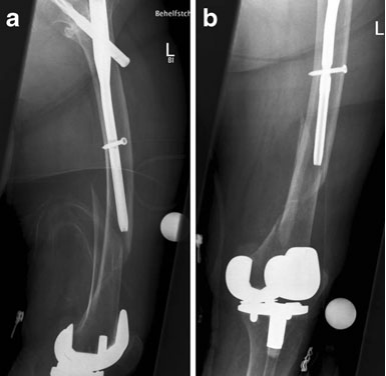
Anteroposterior (**a**) and lateral (**b**) view of a Rorabeck type 2 and OTA 32-A1 fracture around a resurfacing TKA and a proximal femur Nail (PFN A) in an 87-year-old female patient; by Florian Gebhard

**Fig. 6:**
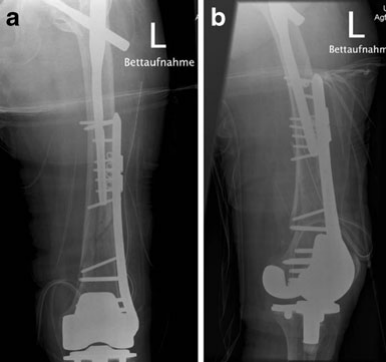
Postoperative anteroposterior (**a**) and lateral (**b**) views after open fixation with a monoaxial locking plate. For fixation around the intramedullary implant, a locking attachment plate (Synthes®) was applied

Although monoaxial locking plates usually provide high primary stability, resisting high pull out forces, the application in situations with an intramedullary implant may be difficult. Therefore, additional devices like cerclage or additional plates (e.g. locking attachment plate; Synthes®) have to be applied at the level of the prosthesis to provide stable plate fixation (Figs. 5 and 6).

##### Polyaxial locking plates

**Fig. 7:**
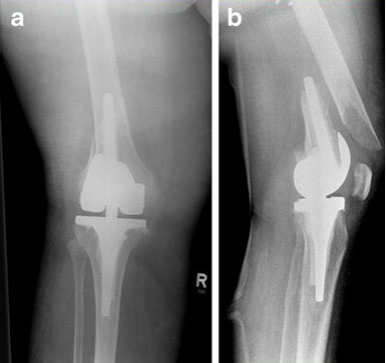
Anteroposterior (**a**) and lateral (**b**) view of a Rorabeck type 2 and OTA 33-A1 fracture of a hinged TKA in a 76-year-old female patient. Because of the stem and the box, there is only limited bone stock in the distal femur (**b**); by Steffen Ruchholtz

**Fig. 8:**
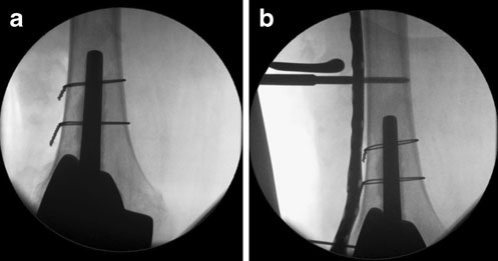
Intraoperative pictures demonstrating the Mini open technique of open reduction with cerclage fixation (**a**) and stabilisation by a periprosthetic polyaxial locking plate (NCB PP; Zimmer®; **b**). Three distal locking screws were placed around the stem (**c**)

**Fig. 9:**
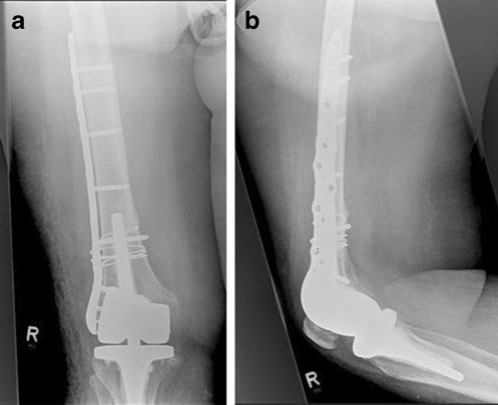
**a, b** Follow-up X-ray pictures at 6 months after surgery. Because of the fixation with three distal screws, one additional cerclage was set around stem and plate

**Fig. 10:**
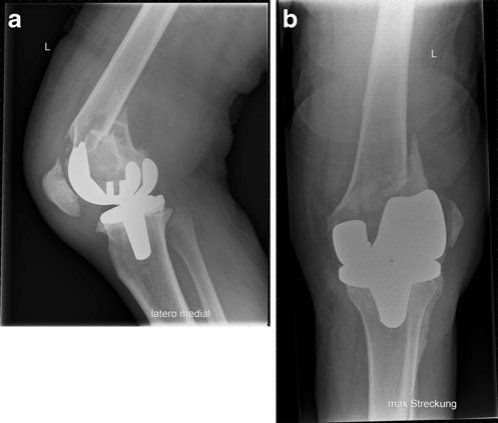
Anteroposterior (**a**) and lateral (**b**) view of an interprosthetic Rorabeck type 2 and OTA 33-A 3 in an 87-year-old man; by Steffen Ruchholtz

**Fig. 11:**
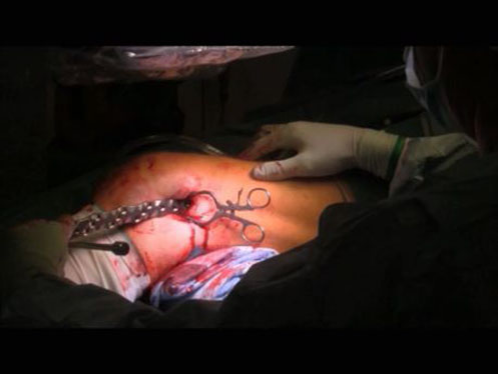
Intraoperative picture of a minimally invasive insertion of a periprosthetic plate (NCB PP)

**Fig. 12:**
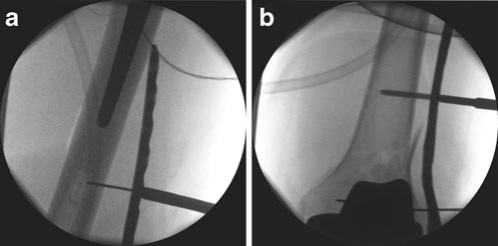
Intraoperative X-ray pictures demonstrating the minimally invasive technique of closed reduction with temporary proximal plate fixation (**a**) and reduction of the fragments by the screws through the plate (**b**)

**Fig. 13:**
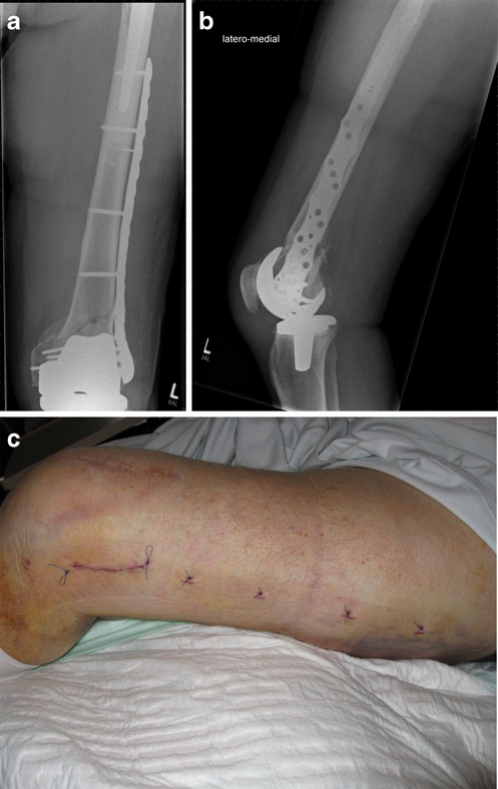
Postoperative anteroposterior (**a**) and lateral (**b**) views. The plate was selected to be long enough to over lap the tip of the THA in order to prevent stress raising at the ‘inter implant’ region. **c** shows the dimension of the needed incisions

In order to pass by an intramedullary implant, polyaxial locking screws (e.g. NCB® - System; Zimmer®) might be of advantage. These implants offer the possibility of a polyaxial locking screw fixation in up to 15° in any direction to the plate level (full range of 30°). Some designs allow a reduction of the fragments in direction to the plate by the screws before they are locked. With the NCB®-System, angular stability is achieved by fixing the head of the screw with an additional cap turned into the plate (Figs. 7, 8, 9, 10, 11, 12, and 13).

Specially designed periprosthetic plates (NCB-PP®; Zimmer®) have a broader metaphyseal area augmenting the possibilities in PPF fixation especially around uncemented implants (Fig. 8c)

#### Surgical technique

Plating may be performed in different techniques of reduction and fixation. Reduction can be achieved either directly (forceps and lag screw/cerclage) in two-part long spiral fractures (OTA Type 32 or 33-A1) or by bridging the fracture zone when direct reduction cannot be achieved (e.g. multi-fragmented fractures). These principles should be respected independently from the selected approach (see below).

Although ‘open’ techniques allow for direct visualisation of the fracture, impairment of the local bony perfusion after manipulation of the soft tissue is of some concern. Since fracture healing is already impaired in geriatric patients, especially when there is an intramedullary implant, soft tissue-preserving strategies like a ‘mini open’ or a ‘minimally invasive’ technique might be of certain advantage.

The ‘open’ technique is a common procedure for PPF fixation. Using a lateral subvastus approach after ligation of the perforator vessels, the bone is exposed. The incision has to be long enough to allow for the application of a plate that is sufficiently long (Figs. 5 and 6). Bony fragments have to be managed with care to avoid impairing the soft tissue that provides the local blood supply.

The ‘mini open’ technique is an alternative in two-part spiral fractures classified as OTA (Orthopeadic Trauma Associoation) type 32-A1 or 33-A1. For this technique, an incision at the level of the plate insertion is made, sufficiently long to expose the fracture region. The two fragments are reduced by the help of a forceps until an optimal contact with anatomical alignment of axis and rotation is achieved. The reduction forceps is then replaced by cerclages or a lag screw (Figs. 7, 8, and 9).

After this step, the plate is inserted and temporarily fixed percutaneously with K-wires proximally and distally. Before the screws are set, a lateral view to control the plate position is performed by use of the intensifier.

The screws in the diaphyseal region are inserted percutaneously. The femur is not exposed in the diaphyseal area.

The concept of the ‘minimally invasive’ technique is a totally closed reduction. Reduction is achieved by either ligamentotaxis and/or the application of the plate as a template. Therefore, maintaining the correct alignment by axial traction throughout the whole procedure is of essential priority. Traction can be exerted throughout the procedure by the assistant surgeon. After closed reduction, the plate is inserted on the level of the prosthesis after a short 3- to 4-cm incision (Fig. 11). After this step, the plate is temporarily fixed with K-wires proximally and distally, length must be restored at this point (Fig. 12). Before the screws are set, a lateral view to control the plate position is performed with the intensifier.

By setting the shaft screws, the plate can be used as reduction tool (Fig. 12) if no primary locking screws are applied. Locked screws can be set when the plate is running parallel to the diaphysis.

Before the screws are placed in the metaphyseal area, the axis has to be controlled. Some institutions use the ‘cable-technique’ where the straightened cable of the electric coagulation device simulates the mechanical axis. Correct reduction is achieved when the intensifier proves that the straightened cable is projected on the centres of hip, knee and ankle. Thereafter, the screws are set in the metaphyseal region (Fig. 13).

#### Revision prosthesis

**Fig. 14:**
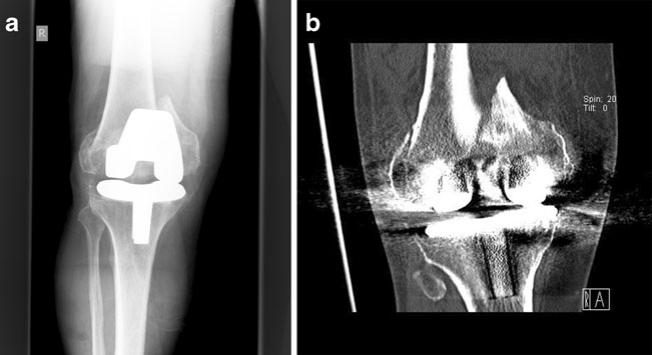
Preoperative X-ray (**a**) and CT (**b**) view of a partially loose PPF type Rorabeck III, OTA 33 C1; by Steffen Ruchholtz

**Fig. 15:**
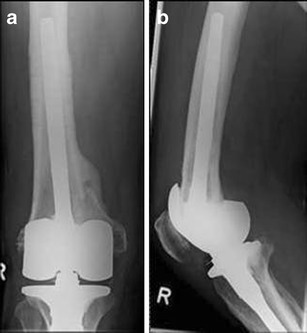
**a, b** Postoperative x-ray after implantation of a hinged revision TKA

n patients with prosthetic loosening, the whole implant has to be exchanged to revision prosthesis with diaphyseal fixation (Figs. 14 and 15). For revision, mostly modular systems are applied that are implanted by a lateral or standard parapatellar incision (in short distal fragments). In general, a hinged prosthesis has to be implanted because of the involvement or the resection of the collateral ligaments. Therefore, the whole implant including the tibial component has to be exchanged (Fig. 15) in most cases. In patients with severe bone loss around the implant, partial replacement of the distal femur (e.g. resection prosthesis) may be considered.

## Tibia

Periprosthetic fractures around the tibial component are rare, with an incidence of <4 %.

### Diagnostics

Analogous to fractures around the femoral component, the stability of the prosthesis is evaluated with respect to the patients’ history before trauma and X-ray diagnostics. Typical signs of loosening like displacement of the shield from the distal femur or separation of cement may be seen on x-ray in cases of prosthesis loosening. Nevertheless, in difficult cases, a CT-scan may be helpful in order to detect loosening around the tibial component.

### Classification

Felix et al. have suggested a classification based on a study on 102 tibial fractures around total knee implants [8].

Type 1: Fracture partially involving the tibial head
Type 2: Fracture involving the whole tibial head around the implant
Type 3: Fracture lying below the distal part of tibial component
Type 4: Fracture with an isolated involvement of the tuberosity

The four fracture types in the classification are combined with a suffix:

- A—stable prosthesis
- B—loose prosthesis
- C—intraoperative fracture

### Conservative treatment

Fractures without displacement around a stable prosthesis may be treated conservatively. This strategy is recommended for most undisplaced partial fractures of the tibial head (Type 1A and 1C fracture). Fractures involving the whole tibial head may be treated conservatively, particularly when they occur intraoperatively during the implantation of the prosthesis and do not show any displacement in neither of the planes on X-ray (Type 2A and 2C fracture). Conservative treatment includes cast fixation and non-weight bearing of the knee for 6 weeks.

### Surgical treatment

**Fig. 16:**
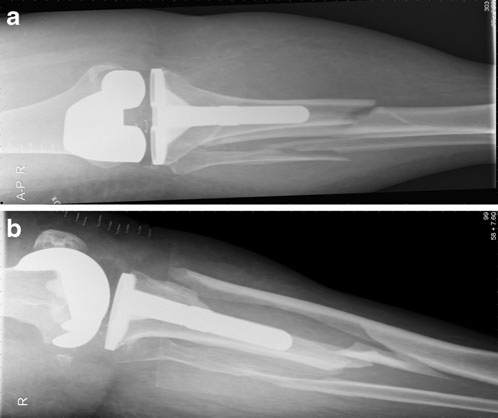
**a, b** Preoperative X-ray of a periprosthetic fracture around the tibial component of a TKA type Felix 3A; by Morten Schultz Larsen

**Fig. 17:**
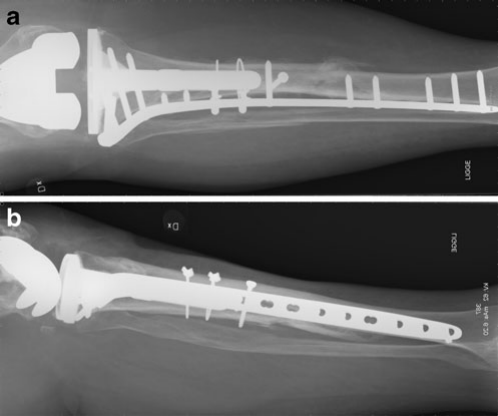
**a, b** Postoperative X-ray 6 months after stabilisation with a locking plate (LISS; Synthes®)

Surgical revision is recommended in all displaced tibial PPFs. Even undisplaced fractures below the prosthetic component (Type 3A or 3C) should be considered for internal fixation because of a high risk for pseudarthrosis (Figs. 16 and 17).

All types of fractures that are accompanied by a loosening of the tibial component should be treated by a revision of the implant.

#### Plates

For fractures with a stable implant (types 2A and C; types 3A and C), monoaxial or polyaxial locking plates are the treatments of choice. They can be applied either in open or in a less invasive technique. Because of the relatively thin soft tissue layer, percutaneous fixation of the distal plate to the diaphysis after ‘mini open’ or ‘minimally invasive’ reduction is easy to achieve and allows a maximum preservation of the local blood supply.

The risk of soft tissue complication, including deep infection is much higher than on the distal femur, and the surgeon must have this in mind when planning the treatment. Fractures of the tuberosity (type 4) may be fixed either by isolated lag screws (big fragments) or by plates.

#### Revision prosthesis

In a loose prosthesis (fractures with a suffix B), the whole implant has to be exchanged to revision prosthesis with diaphyseal fixation. For revision, modular systems are applied that are implanted by a lateral or standard parapatellar incision (in short proximal fragments). In general, a hinged prosthesis should be implanted because of the involvement or the resection of the collateral ligaments. Therefore, the whole implant including the femoral component has to be exchanged.

In cases with severe bone loss around the implant, partial replacement of the proximal tibia (e.g. tumour prosthesis) should be considered.

## Postoperative care

It is of great importance for the aged patient to be mobilised out of bed early in order to prevent the health problems of immobilisation. Nevertheless, primary stability that allows full weight bearing cannot always be achieved by plating or nailing. In most of the cases, partial weight bearing is recommended using the aid of frames during the first 6 weeks after surgery.

In well-fixed revision prostheses with a cemented stem, early full weight bearing might be allowed. In uncemented revision stems, care must be taken out within the first 6 weeks after the operation. After consolidation of the soft tissue, all PPF surgery patients should be treated with continuous passive motion of the knee.
